# Specific Activation of Dendritic Cells Enhances Clearance of *Bacillus anthracis* following Infection

**DOI:** 10.1371/journal.pone.0109720

**Published:** 2014-11-07

**Authors:** Iain J. T. Thompson, Elizabeth R. Mann, Margaret G. Stokes, Nicholas R. English, Stella C. Knight, Diane Williamson

**Affiliations:** 1 Biomedical Sciences Department, Defence Science and Technology Laboratory, Porton Down, Salisbury, Wiltshire, SP4 0JQ, United Kingdom; 2 Antigen Presentation Research Group, Imperial College London, Northwick Park and St. Mark's Campus, Watford Road, Harrow, HA1 3UJ, United Kingdom; Auburn University, United States of America

## Abstract

Dendritic cells are potent activators of the immune system and have a key role in linking innate and adaptive immune responses. In the current study we have used *ex vivo* pulsed bone marrow dendritic cells (BMDC) in a novel adoptive transfer strategy to protect against challenge with *Bacillus anthracis*, in a murine model. Pre-pulsing murine BMDC with either recombinant Protective Antigen (PA) or CpG significantly upregulated expression of the activation markers CD40, CD80, CD86 and MHC-II. Passive transfusion of mice with pulsed BMDC, concurrently with active immunisation with rPA in alum, significantly enhanced (p<0.001) PA-specific splenocyte responses seven days post-immunisation. Parallel studies using *ex vivo* DCs expanded from human peripheral blood and activated under the same conditions as the murine DC, demonstrated that human DCs had a PA dose-related significant increase in the markers CD40, CD80 and CCR7 and that the increases in CD40 and CD80 were maintained when the other activating components, CpG and HK *B. anthracis* were added to the rPA in culture. Mice vaccinated on a single occasion intra-muscularly with rPA and alum and concurrently transfused intra-dermally with pulsed BMDC, demonstrated 100% survival following lethal *B. anthracis* challenge and had significantly enhanced (p<0.05) bacterial clearance within 2 days, compared with mice vaccinated with rPA and alum alone.

## Introduction

Dendritic cells (DCs) are potent activators of the adaptive immune system [1] and are critical for host defence against pathogens. Naïve DCs perform a surveillance function, constantly sampling their environment, taking up antigen by phagocytosis and with increased efficiency by receptor-mediated endocytosis [2]. Upon maturation, DCs alter their phenotype and home to lymph nodes where they initiate and polarise an adaptive immune response by presentation of peptides on MHC-II molecules to CD4^+^ T cells or by cross-priming on MHC-I molecules to CD8^+^ T cells [3]. Their ability to induce antigen-specific T cell and antibody responses and their ability to be easily cultured *in vitro* has enabled DCs to be trialled as cellular vaccines, both for infectious diseases and in cancer, resulting in either tumour or infectious disease regression or eradication [4]. However, the passive transfusion of activated DC raises logistic and practical difficulties for clinical use. An alternative approach is to activate DCs *in situ* through the ligation of their cell surface receptors with monoclonal antibody conjugated to the antigen of interest, which has been shown to be an efficacious method of enhancing adaptive immune responses to pathogens [5].

*Bacillus anthracis*, the etiological agent of anthrax, is a Gram-positive spore-forming bacterium. The disease has three clinical presentations dependent on the route of inoculation, cutaneous, pulmonary and gastrointestinal anthrax. The sporulating nature of *B*. anthracis confers survival advantages in the environment and enables infection and persistence *in vivo*. Spores can exploit host cells to persist in niches and evade host immune responses capable of bacterial clearance, before germinating into vegetative cells. *B. anthracis* possesses two virulence plasmids: pXO1, encoding the proteins protective antigen (PA), lethal factor (LF) and edema factor (EF) which can form the binary toxins Lethal Toxin (LT) and Edema Toxin (ET) [6]; and pXO2, encoding the poly-D-glutamic acid capsule [6] required for immune evasion and intracellular survival [7].

Vaccines are an important strategy to protect at-risk individuals. Licensed anthrax vaccines such as Anthrax Vaccine Precipitate (AVP) and Anthrax Vaccine Adsorbed (AVA), together with next generation anthrax vaccines such as rPA and alum, require several priming doses followed by annual boosters. They protect against disease principally by inducing high titre antibody to PA, which neutralises anthrax toxins in the early stages of infection [8], [9], [10]. Despite the ability to induce a high titre antibody response, vaccines comprising alum as an adjuvant are generally poor inducers of cell-mediated immune (CMI) responses and enhanced CMI responses through DC vaccination may improve vaccine protective efficacy [11].

In this study we have explored the synergistic effects of passively administering specifically-activated DC at the same time as actively immunising mice with rPA and alum, to determine whether this will significantly reduce the time required to develop protective immunity against anthrax. In addition to survival, a key endpoint of this study was to determine whether this approach significantly enhanced bacterial clearance from the spleen in vaccinated and challenged mice and whether human DC would respond to activation in the same manner qualitatively and quantitatively as murine DC.

## Results

### Activation of DCs

**Figure 1:**
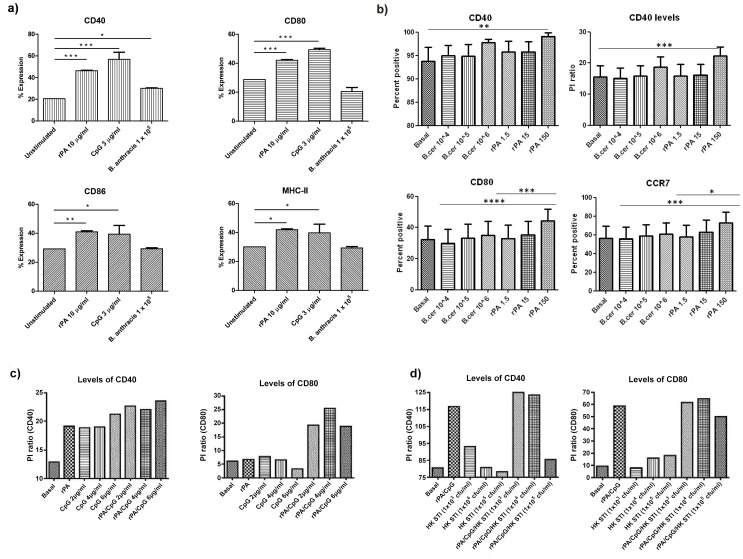
Co-stimulatory molecule expression on DC following antigen pulsing. **a**) Upregulation of MHC-II and co-stimulatory molecules on murine BMDCs. Costimulatory molecules on DCs were routinely upregulated by rPA and CpG following overnight co-culture whilst heat killed *B. anthracis* induced weak responses. Samples were analysed using a one-way-Anova with a Tukey post-hoc test (* p<0.05, ** p<0.01, *** p<0.001). **b**) Upregulation of CD40 and CD80 on human DC with rPA. Graphs represent mean±SEM proportion of DC expressing CD40 (n = 7), CD80 (n = 10) and CCR7 (n = 8). Levels of CD40 expression are also represented by mean±SEM positive intensity (PI) ratio (n = 7). One-way ANOVA (repeated measures) with ad-hoc Bonferroni corrections was applied (*p<0.05, **p<0.01, ***p<0.001, ****p<0.0001). **c**) Confirmatory experiments representing effects of rPA/CpG combined on levels of CD40 and CD80 expression, compared to rPA alone (and basal conditions/medium only). **d**) Confirmatory experiments representing effects of combination of vaccine antigens (rPA and HK *B. anthracis*) with CpG compared to rPA/CpG combination (n = 2).

The activation status of DCs was investigated prior to their use in transfusion. DCs were stimulated *ex vivo*, with vaccine antigens (rPA or heat-killed (HK) *B. anthracis* spores) or adjuvant (CpG) and the upregulation of costimulatory markers was assessed by flow cytometry. Pulsing of murine BMDC with rPA significantly upregulated (p<0.001) the expression of CD40, CD80, CD86 and MHCII, as effectively as pulsing with CpG. In contrast, HK *B. anthracis* caused the significant upregulation (p<0.05) of CD40 only on DC (Figure 1a).

In parallel with the studies on murine BMDC, *ex vivo* human DC, expanded from normal human peripheral blood mononuclear cells (PBMC), were pulsed with vaccine antigens and/or adjuvant, to determine the effect on their maturation and activation status. In these human studies, heat-killed *Bacillus cereus* was used in addition to HK *B. anthracis* as a positive control, since *B. cereus* is a related bacillus which causes gastrointestinal food-borne illness. Optimisation experiments to determine rPA (and *B. cereus*) doses demonstrated that similar to murine BMDC, pulsing of human DC with rPA significantly upregulated CD40 (p<0.01) and CD80 (p<0.001) (Figure 1b). Lymph-node homing marker CCR7 (upregulated on DC by maturation and activation) was also upregulated by rPA (p<0.001; Figure 1b). There was no change in expression of costimulatory markers CD83 or CD86 or in expression of receptors involved in DC recognition of bacteria or bacterial products (including Toll-like receptors 2 and 4; data not shown). Using an optimised rPA dose of 150 ng/ml for pulsing human DC, in combination with CpG, resulted in enhanced levels of CD40 and CD80 compared to rPA alone (Figure 1c), and the addition of HK *B. anthracis* to this combination enhanced expression even further (Figure 1d).

### Cellular immune response to DC vaccination

**Figure 2:**
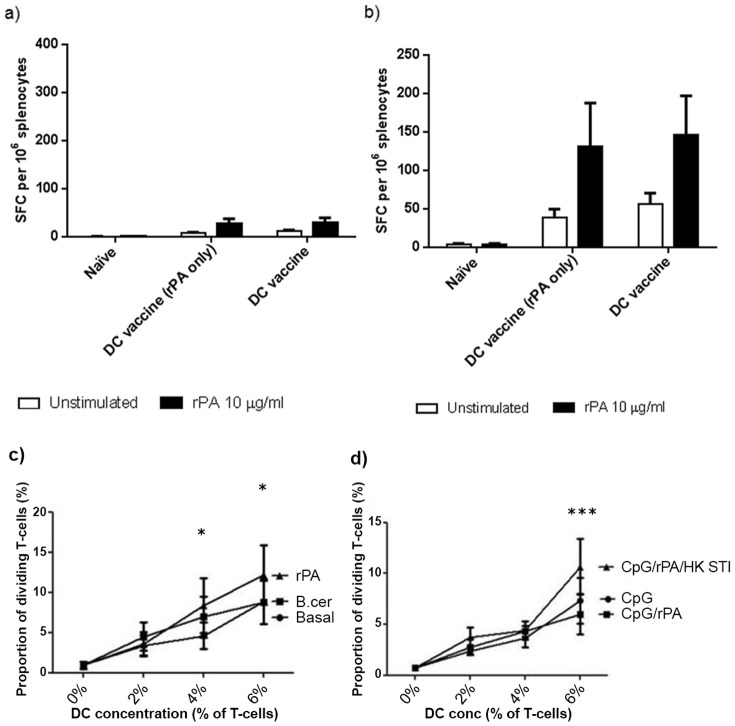
Stimulation of T cell responses. **a and b**) DCs were stimulated with rPA only (DC vaccine rPA only) or with PA, heat killed *B. anthracis* and CpG oligonucleotides (DC vaccine) and administered i.d. to A/J mice. Spleens were taken at 7 days (a) and 14 days (b) post- vaccination and restimulated *ex vivo* with rPA. A one-way Anova with Tukey multiple comparison post-hoc test was performed on the results (* p<0.05, ** p<0.01, *** p<0.001). Error bars represent the standard error of the mean calculated from the means of three replicates from five animals. **c**) Proliferation of CD4^+^ naive human T cells following 5-day co-culture with syngeneic human DC pulsed with medium only, *B. cereus* or rPA (n = 5). After 2-way ANOVA analysis with Bonferroni corrections, there were no significant differences between *B. cereus* and medium only (basal)-pulsed DC regarding stimulatory capacity but rPA-pulsed DC were significantly more stimulatory than basal conditions at 4% and 6% (p<0.05 in both cases) and significantly more stimulatory than *B. cereus* pulsed DC at 6% (p<0.05). **d**) CD4^+^ naive human T-cell proliferation following 5-day co-culture with syngeneic DC pulsed with CpG only, rPA with CpG or combination of rPA, CpG and HK *B. anthracis* (n = 3). After 2-way ANOVA with Bonferroni corrections, there were no significant differences between CpG and rPA/CpG-pulsed human DC regarding stimulatory capacity but rPA/CpG/HK *B. anthracis*-pulsed human DC were significantly more stimulatory than both other conditions at 6% (p<0.001 in both cases).

The cellular immune response of mice transfused with BMDC pre-pulsed with rPA only (DC vaccine rPA only) or BMDC pre-pulsed with rPA, HK *B. anthracis* and CpG (DC vaccine), was characterised using an IFNγ ELISPOT to measure the recall response of their splenocytes *ex vivo* for rPA. Whilst an increase in the number of IFNγ SFC per 10^6^ splenocytes was observed between sample days 7 (Figure 2a) and 14 (Figure 2b) for groups transfused with either unstimulated DC or either of the DC vaccines, this increase was not rPA-specific as there was no significant difference in number of IFNγ SFC between splenocyte samples which were rPA stimulated *ex vivo*, or unstimulated, at either time-point.

### Pulsed human DC can activate naïve T-cells *ex vivo*

For comparison, *ex vivo* human DC pre- pulsed with either rPA or with *B. cereus* as a positive control, were cultured with naïve human T cells to determine if the latter could be specifically activated. Human naive (CD45RO^−^) CD4^+^ blood T cells were enriched from normal PBMC and were CFSE- labelled prior to co-culture with rPA, HK *B.anthracis* (STI strain) and/or CpG-pulsed DC, from the same donor in a syngeneic T cell stimulation assay. Human DC induced a dose-dependent proliferation of naive CD4^+^ T cells in all cases. In initial experiments, rPA-pulsed DC stimulated a significantly enhanced proliferation of T cells compared with *B. cereus* or T cells stimulated by DC pulsed with medium only (p<0.05; Figure 2c). In later experiments, following optimisation of combination doses of vaccine antigens and adjuvant, the combination of rPA, CpG and HK *B. anthracis* (which we previously demonstrated induced the highest expression of costimulatory molecules on DC) was used to pulse DC and stimulated a significantly greater proliferation of naïve T cells (p<0.001) than DC pulsed with adjuvant (CpG) only or CpG/rPA combined (Figure 2d).

### Cytokine production by dividing human T-cells

**Figure 3:**
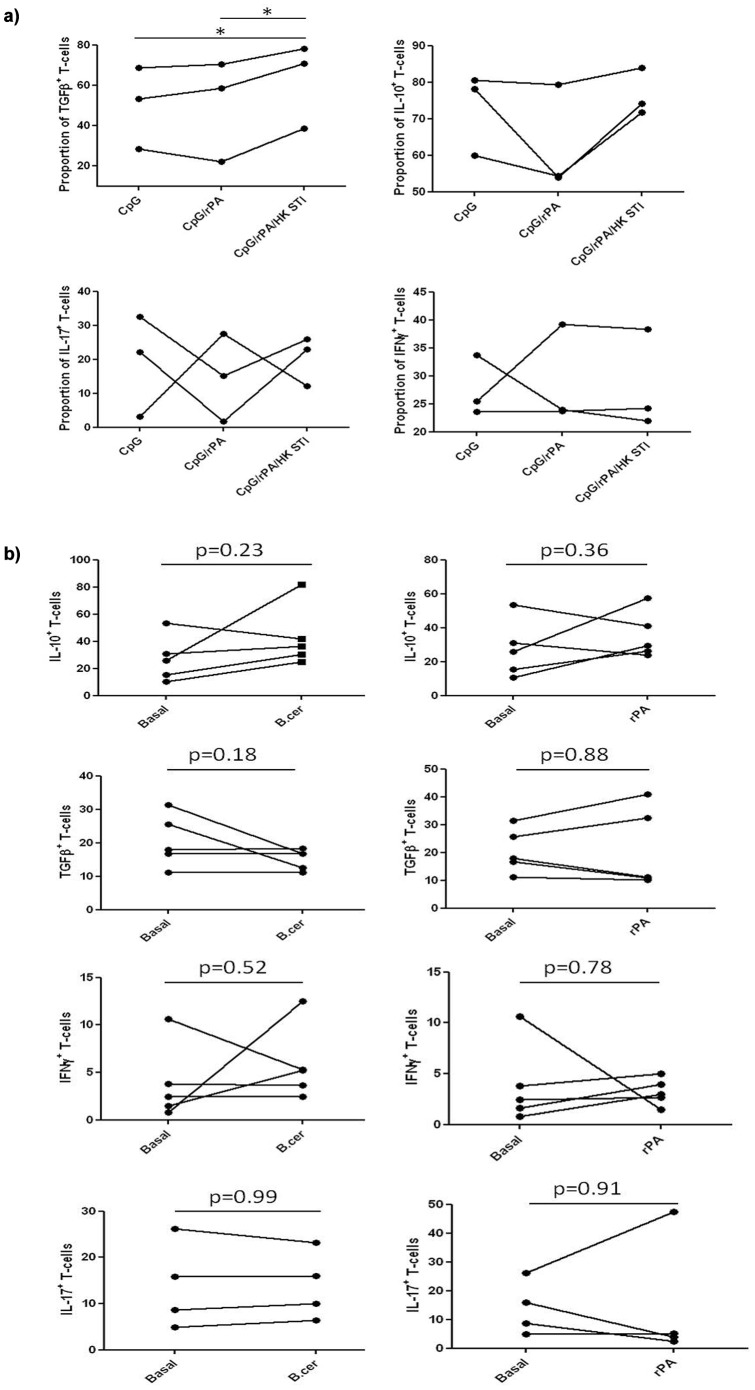
Cytokine production by human T-cells. Cytokine production by human T-cells stimulated by 6% medium (basal) or *B.cereus* or rPA-pulsed syngeneic DC (n = 5) (Panel A) or by 6% CpG-, rPA/CpG- or rPA/CpG/HK STI-pulsed syngeneic DC (n = 3) (Panel B) following 5-day co-culture. Paired t-test was applied, p<0.05 was considered statistically significant (*p<0.05, **p<0.01, ***p<0.001).

Cytokine production by human T-cells dividing in response to a 5-day co-culture with syngeneic DC pulsed variously with rPA plus CpG, or rPA plus CpG plus HKSTI, was determined (Figure 3). No significant differences in T-cell cytokine production were seen upon stimulation with rPA-pulsed DC compared to medium only (Panel A), but T-cells stimulated with rPA/CpG/HK STI-pulsed DC produced significantly more TGFβ than those stimulated with rPA/CpG- or CpG only-pulsed DC (Panel B). There was no significant change in production of other cytokines studied (IL-10, IL-17 or IFNγ).

### Cellular immune response to DC vaccination in combination with active immunisation

**Figure 4:**
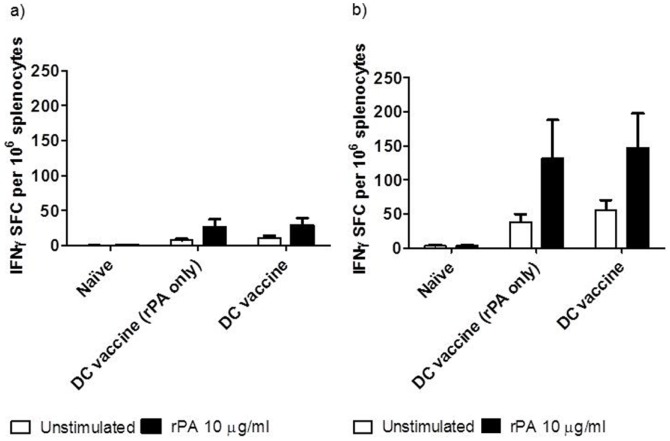
IFNγ spot- forming cells as measured by ELISPOT. DCs were stimulated with rPA, heat killed *B. anthracis* and CpG oligonucleotides and administered i.d. in combination with rPA and alum given i.m to A/J mice. Spleens were taken seven and fourteen days post vaccination and restimulated with rPA. A one-way Anova with Tukey multiple comparison post-hoc test was performed on the results (* p<0.05, ** p<0.01, *** p<0.001). Error bars represent the standard error of the mean calculated from the means of three replicates from five animals.

When splenocytes prepared from immunised mice were re-stimulated *ex vivo* with rPA, there was a significant increase in IFNγ^+^ SFC from mice actively immunised with rPA and alum compared to naïve mice at days 7 (p<0.05; Figure 4a) and 14 (p<0.001; Figure 4b). The recall response to rPA at the earlier time point of 7 days post-immunisation, was even further enhanced in mice actively immunised with PA and alum together with transfusion of DC vaccine (p<0.001; Figure 4a). By day 14, both the actively immunised (rPA and alum) and the combined immunisation groups (rPA and alum and DC vaccine) had a significantly elevated (p<0.01 and p<0.001, respectively) recall response compared with mice given unstimulated DC, with no significant difference between the two immunised groups.

### Anti-PA response to DC vaccination in combination with active immunisation

**Figure 5:**
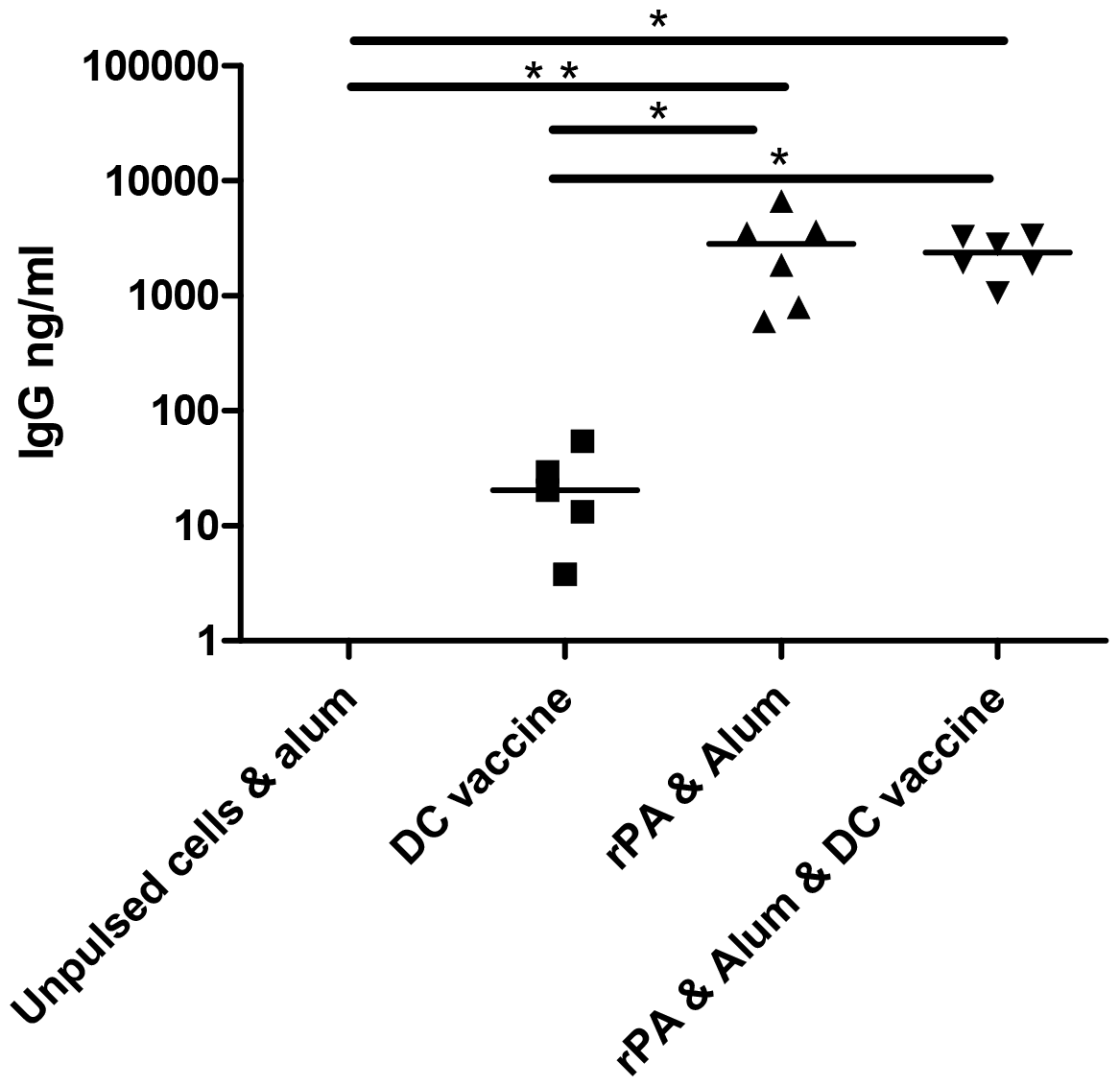
Antibody responses 14 days after either stimulated DCs, or rPA and alum, or both, were administered. Each point represents the mean of three replicates from five animals. A one-way Anova with Tukey multiple comparison post-hoc test was performed on the results (* p<0.05, ** p<0.01, *** p<0.001).

Analysis of day 14 sera from mice that had been transfused with DCs pre-pulsed with rPA, CpG and HK *B. anthracis* showed that they had developed a specific IgG response to PA (Figure 5). Mice which had been immunised with rPA and alum, or with rPA and alum and also transfused with the DC vaccine, had significantly elevated (p<0.05) anti-rPA IgG titres, when compared with mice receiving stimulated DCs alone (DC vaccine). In terms of antibody induction however, the combination of active immunisation with passive transfusion of pulsed DC, had no additive effect. In pilot studies we observed that the predominant IgG sub-class induced in mice either actively immunised with rPA & alum or adoptively transfused with rPA-pulsed DC, or both actively and passively immunised, was IgG1 (data not shown).

### Post-challenge bacteriology and survival

**Figure 6:**
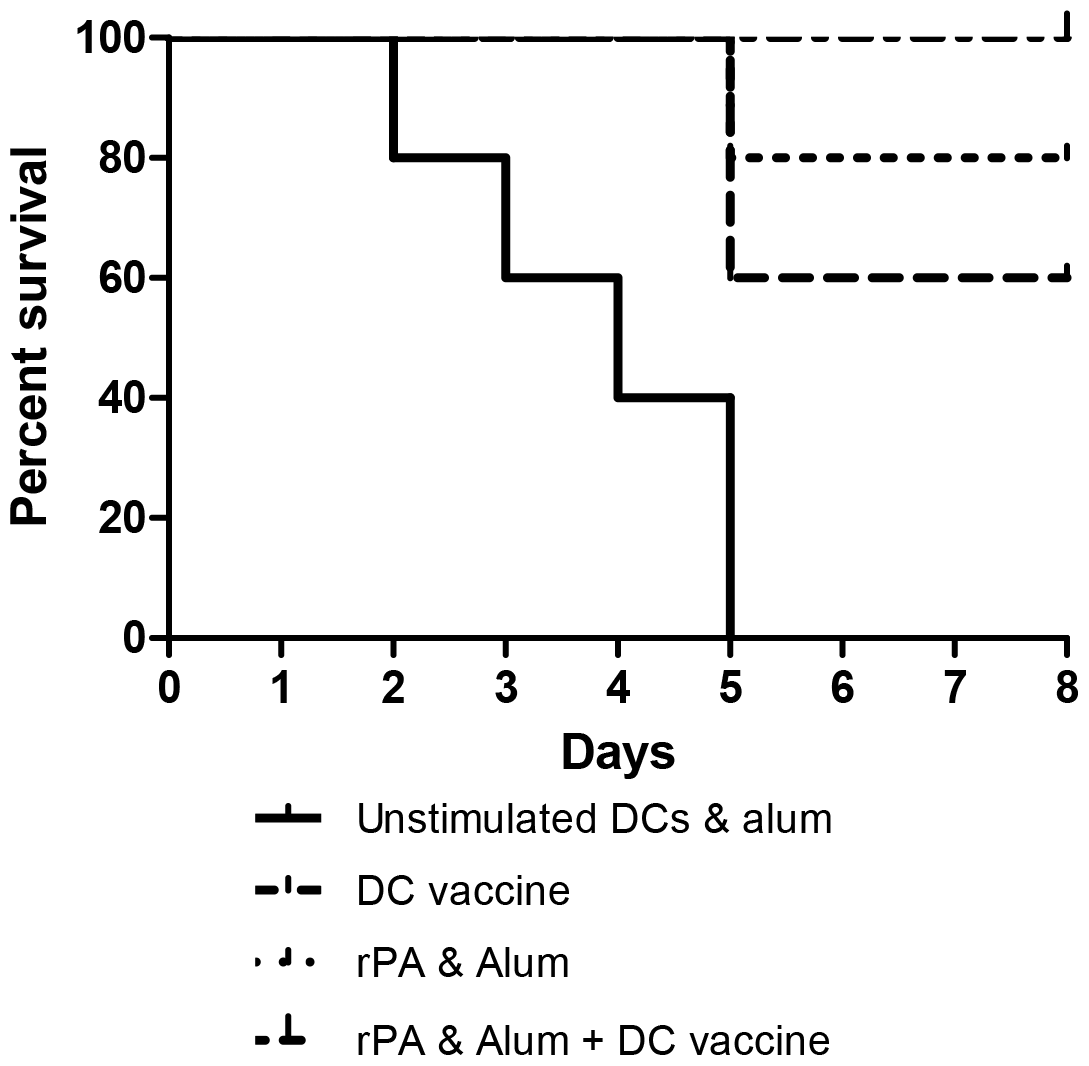
Post Challenge survival. Groups of 5 A/J mice immunised with a DC vaccine (comprising of DCs stimulated overnight with rPA, heat-killed *B. anthracis* and CpG), rPA in alum, or rPA in alum plus DC vaccine, or left naïve, were challenged at 14 days post-immunisation with 3×10^4^ CFU *B. anthracis* STI (i.p.) and monitored for survival over the subsequent 8 days. There was a statistically significant difference in the survival curves for the negative control (alum+BMDC&CpG) group compared with the group actively immunised with rPA& alum and also receiving rPA&HK *B.anthracis*-pulsed DC (p<0.002) by both the Mantel-Cox and Gehan-Breslow-Wilcoxon tests. Comparison of survival curves between the negative control (alum+BMDC&CpG) group and the group receiving rPA&HK *B.anthracis*-pulsed DC only, was also statistically significant (p<0.01) when both tests were applied.

**Figure 7:**
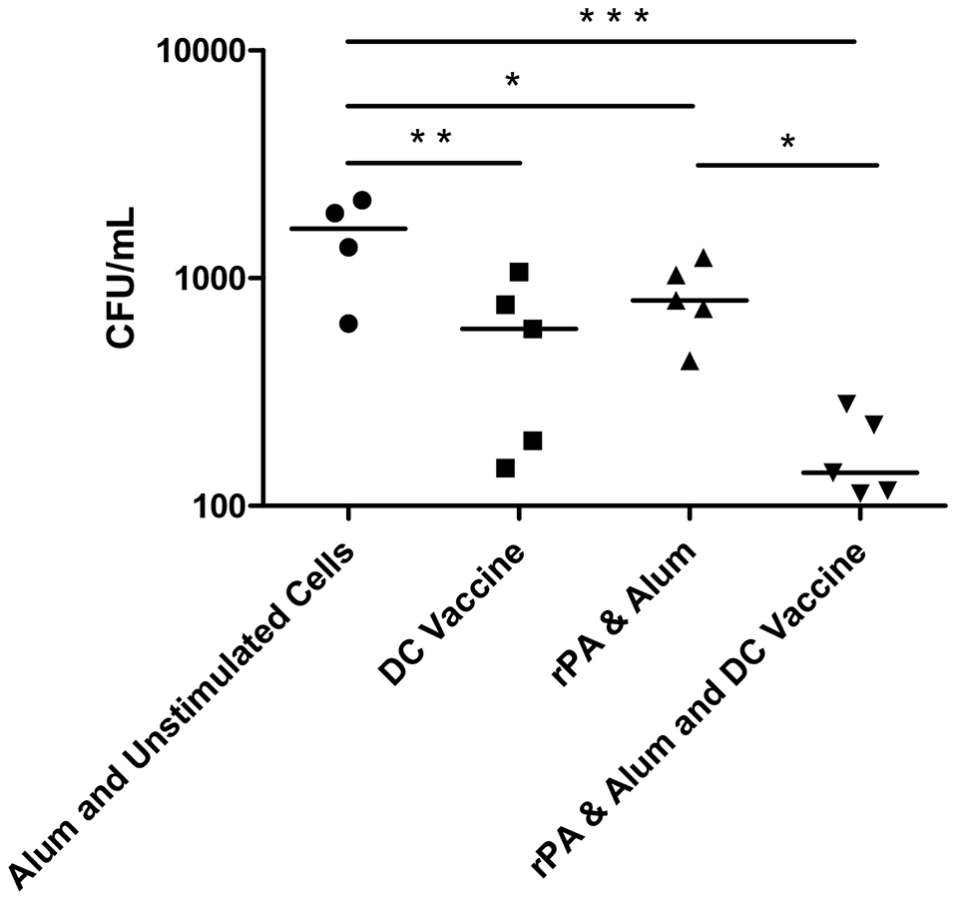
Enumeration of viable *B. anthracis* in the spleens of cohorts of mice two days post challenge. Each point represents the mean of three replicates from five animals, four from the naïve group due to an early death. A one-way Anova with Tukey multiple comparison post-hoc test was performed on the results (* p<0.05, ** p<0.01, *** p<0.001).

Mice were challenged with 3×10^4^ CFU *B. anthracis* STI i.p. (approximately 10 median lethal doses) 14 days post-immunisation. Mice treated with PBS only had a mean time to death of 3 days following challenge with 10MLD *B.anthracis* STI (data not shown). Two days post-challenge, a cohort of mice from each treatment group was culled with spleens taken to ascertain bacterial loads. Mice receiving unstimulated DCs together with alum showed limited survival (Figure 6) with all animals dead within 5 days and a splenic bacterial load significantly greater than all other groups (Figure 7). Mice receiving only the DC vaccine had 60% survival but a significantly (p<0.01) reduced bacterial load compared with the group receiving unstimulated DC with alum (negative controls), whilst mice immunised with rPA and alum showed 80% survival with a significantly (p<0.05) reduced bacterial load compared with negative controls. However, mice immunised with both rPA and alum and transfused with the DC vaccine had 100% survival and a highly significant reduction in bacterial load within 2 days of challenge, compared to the negative controls (p<0.001) and to the rPA and alum group (p<0.05).

There was a statistically significant difference in the survival curves for the negative control (alum+BMDC&CpG) group compared with the group actively immunised with rPA & alum and also receiving rPA&HK *B.anthracis*-pulsed DC (p<0.002). Comparison of survival curves between the negative control (alum+BMDC&CpG) group and the group receiving rPA&HK *B.anthracis*-pulsed DC only, was also statistically significant (p<0.01).

## Discussion

Whilst there are currently licensed prophylactic vaccines for anthrax (e.g. Anthrax Vaccine Adsorbed, AVA and Anthrax Vaccine Precipitated, AVP) they have several limitations including a long primary schedule that requires multiple vaccinations (0, 3, 6 and 32 weeks currently for the UK AVP) to induce immunity, regular booster doses to maintain protective immunity and relatively poor development of CMI responses capable of clearing spores or vegetative bacteria. Furthermore, prophylactic antibiotic regimens require those suspected to have been exposed to take prolonged antibiotic courses (60 days) with the risk of residual spores germinating after completion of the course and poor compliance to the regimen. Therefore a vaccine that can effectively induce CMI responses capable of reducing vegetative bacterial or spore burden is desirable, potentially by enhancing existing vaccines. In this study we examined the potential for DC transfusion to enhance conventional anthrax vaccination in terms of reducing the time required to develop protective immunity and enhancing the specific CMI response induced, with the rationale that this could significantly improve bacterial clearance post-exposure.

The *in vitro* co-stimulation of DCs with rPA, heat-killed *B. anthracis* and CpG resulted in the maturation of murine DCs with increased expression of MHC-II, CD80 and CD86 needed for presentation of antigen to CD4^+^ T cells and of CD40, required for the co-stimulation of T and B cells. Similarly, human DC responded to rPA pulsing with enhanced CD40 and CD80 expression, which was further enhanced when CpG was added to the rPA stimulus, and even further when both CpG and HK *B. anthracis* were added to rPA.

Although specifically activated, transfusion of these DC into mice did not induce an antigen-specific cellular immune response, as measured by IFNγ secretion following recall with rPA *ex vivo*, within 14 days of vaccination. This was not surprising, since previous studies have demonstrated that DC vaccination required 35 days to induce an antigen-specific anamnestic IFNγ response [12]. To determine if this time span could be reduced, active immunisation with rPA and alum was combined with DC vaccination. Mice receiving this combined immunisation had significantly enhanced CMI responses at only 7 days, as evidenced by a significant increase in the number of IFNγ^+^ spot-forming cells per 10^6^ splenocytes on re-stimulation with rPA, compared to those immunised with rPA and alum alone, demonstrating that DC vaccination both accelerated and enhanced the response to conventional active immunisation.

Furthermore human DC pulsed with rPA, caused a dose-related proliferative response in naïve human CD4^+^T-cells, which was further enhanced when DC were pulsed with CpG and HK *B. anthracis*, in addition to rPA and this was accompanied by a significantly enhanced secretion of TGFβ. The study of human DC-T cell interactions cannot exactly match the murine studies, since it is entirely based on *ex vivo* T-cell stimulation, however these concurrent human studies supported the murine data demonstrating that rPA, HK *B. anthracis* and CpG is an optimum activating combination for the DC. This allowed the optimum DC-activating combination of rPA+CpG+HKSTI to be deployed together with active immunisation in the mouse, to achieve accelerated T- cell mediated immune responses against *B. anthracis*.

The efficacy of the combined immunisation regimen was stringently tested by challenging mice with multiple lethal doses of *B. anthracis* only 14 days after immunisation. Whilst naïve mice rapidly succumbed to infection, 80% of those immunised with rPA and alum survived. The DC vaccine alone protected 60% of mice and this protection was attributed predominantly to the induction of a specific CMI response. By comparison, the DC vaccine, combined with rPA in alum immunisation, fully protected all the mice and these mice had a highly significant reduction in splenic bacteria as early as 2 days post-challenge. Mice receiving either the DC vaccine only, or rPA in alum only, had bacterial loads which although significantly reduced compared with naïve mice, were still elevated compared with the combined vaccine group.

This study provides proof-of-principle that a single transfusion of specifically activated DCs can augment the response to conventional active immunisation, with benefits in terms of accelerating time to immunity, specific CMI, survival and most significantly, clearance of *B. anthracis* in the murine model. Whilst these are significant findings, the passive transfusion of pre-stimulated DCs is not a pragmatic approach to mass vaccination in the clinic. We are pursuing alternative approaches to enhancing CMI through the *in situ* stimulation of DCs in the host, for example by targeting DCs with relevant antigens (such as PA and spore coat proteins) fused to an antibody directed at a DC surface receptor, such as DEC205, an approach that has been demonstrated successful in protecting against *Y. pestis* in mice [5]. Here, we have used activated DCs prophylactically to enhance CMI, but appropriately activated DCs could also be used in a post-exposure context to augment the CMI response in the early phase of infection. In conclusion, the *ex vivo* pulsing of DCs with pathogen-derived material and defined stimulatory antigens with CpG, has induced the maturation of DCs *ex vivo* and is able to direct the development of specific and efficacious T cell responses in an *in vivo* murine model of anthrax.

## Materials and Methods

### Ethics Statement

All animal procedures were performed in accordance with UK legislation as stated in the UK Animal (Scientific Procedures) Act 1986. The Institutional Animal Care and Use Committee approved the Project licence (PPL 30/2488) which was granted on 02/11/2008.

All procedures with human blood samples were performed under Ethics Committee approval (reference 05/Q405/71 entitled ‘Tissue specific immune regulation by dendritic cells in the intestine and other sites’) from the Outer West London Research Ethics Committee (NHS) on 9 March 2010. An amendment to the protocol (Sub Study Version 1.0/dated 29 November 2010) was approved by the NHS on 14 February 2011. This protocol was reviewed by the U.S. Army Medical Research and Materiel Command (USAMRMC), Office of Research Protections (ORP), Human Research Protection Office (HRPO) and found to comply with applicable DOD, U.S. Army, and USAMRMC human subjects protection requirements. All human samples were obtained with written consent from the donor.

### Mice

A/J mice were purchased from Charles River U.K. and held in specific pathogen -free facilities with free access to food and water and allowed to acclimatise for seven days prior to use. There is a positive correlation between the immunising dose of rPA and the protective antibody response when using A/J mice, making them a suitable model for anthrax infection studies [13]. Furthermore, A/J mice are deficient in the complement protein C5, making them susceptible to toxigenic *B. anthracis* [14]. Animals undergoing challenge were held in rigid isolators with both inflowing and out-flowing HEPA filtered air.

### Murine DC culture method

DCs were prepared using a method modified from previous reports [15], [16]. Briefly, bone marrow was flushed from murine femurs and tibias and passed through a 40 µm cell sieve to create a single cell suspension. Bone marrow cells were washed in complete media (RPMI-1640 supplemented with 10% foetal calf serum, 1% penicillin-streptomycin-glutamine and 50 µM 2-mercaptoethanol) and red cells lysed before being washed again. Cells were seeded at 1×10^6^ cells mL and cultured in a fully humidified atmosphere at 37°C with 5% CO_2_; complete media was supplemented with 20 ng/ml of GM-CSF. Media was replaced at day 3 and 5 before loosely adherent cells were harvested at day 8. Cells were confirmed to be >90% CD11c^+^ by flow cytometry and used in stimulation assays.

### Human DC characterisation method

Human blood was collected from healthy volunteers with no known autoimmune or inflammatory diseases, allergies or malignancies, following informed consent (EC number 05/Q0405/71). Peripheral blood mononuclear cells (PBMC) were obtained by centrifugation over Ficoll-Paque plus (Amersham Biosciences, Chalfont St. Giles, UK). Human low density cells (LDC) were obtained following Nycoprep centrifugation of 20 h.- cultured PBMC prior to antigenic pulsing or T-cell stimulation. Human DC were then identified as HLA-DR^+^ lineage cocktail (CD3/CD14/CD16/CD19/CD34) live cells by flow cytometry for analysis of co-stimulatory molecules and cytokine production. LDC used as a DC source are 98–100% HLA-DR^+^, with morphological characteristics of DC (both at optical and electron microscopy) and are potent stimulators of allogeneic naive T cells [17], [18].

### Stimulation of dendritic cells

Human and murine DCs were stimulated with rPA, CpG ODN (Invivogen) and heat killed *B. anthracis* prepared using a method previously reported [19]. Briefly, *B. anthracis* (STI strain) spores were harvested and washed three times by centrifugation before being resuspended in PBS. The spore suspension was inactivated by incubation in a water bath for 2 h. at 90°C with occasional shaking. After inactivation the culture was checked for viability by inoculating 10 mL broths with heat-killed suspension and incubating at 37°C for one week. L-agar plates were subsequently inoculated with the entire broth and incubated for a further 7 days. An absence of growth indicated the spore suspension to be inactivated.

### Flow cytometry to assess cellular activation

Murine antibodies with the following specificities and conjugations were purchased from Biolegend, CD11c-PeCy7, CD80-PE, CD86-APC, CD40-FITC, MHC-II PerCPCy5.5, together with appropriate isotype matched controls. After staining, cells were fixed with 1% paraformaldehyde in 0.85% saline and stored in the refrigerator prior to acquisition on the flow cytometer, within 48 hours.

Human monoclonal antibodies with the following specificities and conjugations were used: TLR2-FITC (TLR2.3), TLR4-FITC (HTA125), CD40-PE (LOB7/6), DC exclusion cocktail-PE-Cy5 (CD3 (S4.1), CD14 (TUK4), CD16 (3G8), CD19 (SJ25-C1), CD34 (581)) were purchased from AbD Serotec (Oxford, UK). CD83-PE (HB15e), CD80-FITC (L307.4), HLA-DR-FITC/APC (G46-6), CD86-FITC (24F), IL-10-APC (JES3-19F1), IL-12-PE (C11.5), IL-6-FITC (MQ2-13A5), IL-17-PE (SCPL1362), IFNγ-APC (25723.11), FoxP3-PE (259D/C7), CD4-PE (RPA-T4), CD3-PE/PeCy5/APC (UCHT1), CD8-APC (SK1), CD45RO-PE (UCHL1), CD45RA-PeCy5 (H1100) were purchased from BD Biosciences (Oxford, UK). CCR7-PE (150503) and TGFβ-PE (IC388P) were purchased from R&D Systems (Abingdon, UK). Appropriate isotype-matched control antibodies were purchased from the same manufacturers. After staining, cells were fixed with 1% paraformaldehyde in 0.85% saline and stored in the refrigerator prior to acquisition on the flow cytometer, within 48 hours.

### Cytokine production by human DC

Intracellular cytokine production by DC was measured following antigenic pulsing of DC via comparison of monensin-treated DC (4 h.) and non-monensin-treated DC (incubated with medium only for 4 h.). Cells were then labelled for surface marker expression using monoclonal antibodies to identify DC as described above, fixed and permeabilised before labelling for cytokines prior to acquisition.

### Enrichment of CD4^+^ naive T cells for human DC stimulation assays

Blood from the same donor as the DC source was used to isolate human CD4^+^ naive T cells. PBMC were depleted of CD14^+^, CD19^+^, HLA-DR^+^, CD45RO^+^ and CD8^+^ cells using immunomagnetic beads following the manufacturer's instructions. Flow cytometric analysis confirmed that>96% of these cells were CD45^+^CD4^+^ T cells (data not shown).

### Human DC stimulation of T-cell assays

Human CD4^+^ naive T cells were CFSE- labelled and incubated for 5 days with enriched syngeneic DC at 2, 4 and 6% (of total T-cell number), providing dose-dependent proliferation of T cells in all cases. Cells were recovered following 5d. culture and CFSE^lo^ proliferating cells were identified, analysed and quantified by flow cytometry.

### Immunisations

DCs were prepared as above. For mouse immunisations, 2×10^6^ DCs were stimulated with 10 µg/mL rPA, 6 µg/mL CpG and 10^4^ CFU/mL heat-killed *B. anthracis* STI for 18 h. at 37°C. Cells were harvested and washed to remove excess antigen and resuspended in PBS. Mice, 5 per group, were immunised intradermally (i.d.) with 1×10^6^ cells resuspended in 100 µl PBS on day 0 (the DC vaccine). Alternatively, mice were immunised with rPA and alum and received 10 µg rPA formulated in 100 µl of 0.26% v/v alum, intramuscularly (i.m.), on d.0. Mice receiving both DCs and rPA and alum, received these formulations at the same time on d 0.

### ELISPOT

The number of rPA - specific IFNγ^+^ splenocytes from naïve and immunised mice was assessed using an ELISPOT assay. Briefly, 7 or 14 d. post-immunisation mice were culled, spleens removed and macerated through a cell sieve. Cells were pelleted and red cells removed using lysis buffer before being washed, counted and seeded at 2×10^5^ cells per well onto ELISPOT plates coated with anti-mouse IFNγ. Cells were stimulated with rPA, Con A or left unstimulated (media only), overnight in a humidified incubator at 37°C, 5% CO_2_. Assay development occurred as per the manufacturer's instructions and plates read using an automated ELISPOT reader. Results are presented as the difference in spot-forming cells per 10^6^ splenocytes, between treatment groups.

### ELISA

The anti-PA antibody titre was determined as previously reported [20]. Briefly, microtitre plates were coated overnight at 4°C with 5 mg ml^−1^ rPA in PBS. Serum samples were double-diluted in PBS containing 1% w/v skimmed milk powder and incubated for 2 h. at 37°C. Binding of serum antibody was detected using horse-radish peroxidise conjugated goat anti-mouse IgG, diluted 1 in 2000 in PBS, and the substrate 2,20-Azinobis (3-ethylbenzthiazoline-sulfonic acid) (1.09 mM ABTS). Titres were presented as the end-point dilution, which gave an absorbance of 0.1 over background. Group means ± standard error of the mean were calculated.

### Challenge

*B. anthracis* spores of the STI strain were diluted to an estimated challenge dose of 1×10^3^ by serial dilution. *B. anthracis* STI is a pX01^+^, pX02^−^ unencapsulated toxigenic strain of *B. anthracis* that has been used previously as a live spore vaccine. Actual challenge dose was calculated by culturing the inoculum on nutrient agar plates for 48 h. Groups of ten mice were challenged intra-peritoneally (i.p.) and monitored, with those showing signs of severe illness humanely culled. Survivors were culled after 8d..

### Bacteriology

Five mice per group were culled for bacteriology 2d. after challenge. Spleens were macerated through a wire mesh into sterile PBS. Splenocyte suspensions were serially diluted and 100 µL of each suspension added to nutrient agar in triplicate. Plates were incubated at 37°C for 48 h. before colony forming units (CFU) were enumerated.

### Statistical analysis

Statistical analysis using paired and unpaired t-test, and one- and two-way Anova was performed using GraphPad Prism as stated in figure legends, with calculation of mean and standard error of the mean (SEM). Samples were analysed using a one-way-Anova with a Tukey post-hoc test (* p<0.05, ** p<0.01, *** p<0.001). For comparison of survival curves, the log-rank Mantel-Cox and the Gehan-Breslow-Wilcoxon tests were applied.
